# 

**Published:** 1886-10

**Authors:** 


### CONTENTS:

**ARTICLE I—Southern Dental Association.... 241**
**" II—Medical Education for Dentists, and Dental Education for Physicians. By Dr. B. H. Catching.... 261**
**" III—Implantation of Teeth and Pericemental Life. By William J. Younger, M. D.... 264**
**" IV—Much or Little Water in Vulcanizing. By George B. Snow.... 273**
**" V—On the Treatment of Pyorrhœa Alveolaris. By J. Henry Whatford.. 276**
**" VI—Bridge Work. By Dr. L. P. Haskell.... 280**

### EDITORIAL, ETC.

**The University of Maryland, Dental Department.... 282**
**Requirements for Graduation in both Medicine and Dentistry in England and Scotland.... 283**

### MONTHLY SUMMARY.

**A Porcelain Filling.... 286**
**Wire Ligatures.... 288**

